# Defecation-Induced Splenic Rupture in a Patient With Portal Vein Thrombosis After Sleeve Gastrectomy: Case Report and Literature Review

**DOI:** 10.7759/cureus.75668

**Published:** 2024-12-13

**Authors:** Samira Y. Zeid, Sara Kuzmanovska, Ekin Su Gorgulu, Hanifi Onalan, Servet Karagul

**Affiliations:** 1 General Surgery, Atlas University, Istanbul, TUR

**Keywords:** defecation, emergent splenectomy, portal vein thrombosis, sleeve gastrectomy, splenic rupture

## Abstract

Sleeve gastrectomy is the most commonly performed bariatric surgery, with a relatively high safety profile. Splenic rupture following portomesenteric vein thrombosis after laparoscopic sleeve gastrectomy is a rare life-threatening complication. A morbidly obese 38-year-old female patient presented with acute onset abdominal pain 13 days after a laparoscopic sleeve gastrectomy. Radiological studies revealed portal vein thrombosis (PVT) and splenic hematoma. On the second day of hospitalization, while under observation with stable vital signs, the patient developed sudden hypotension and tachycardia after defecation. Emergency diagnostic tests revealed a splenic rupture, and an emergency laparotomy and splenectomy were performed. The patient received low-molecular-weight heparin (LMWH) and was discharged uneventfully 10 days after surgery. Patients who develop PVT after bariatric surgery should be closely monitored due to the risk of splenic rupture, especially in situations that cause intra-abdominal pressure changes such as defecation.

## Introduction

Over the past few decades, the number of sleeve gastrectomy (SG) procedures has increased dramatically, and with it the incidence of portomesenteric vein thrombosis. According to the literature reviewed, the incidence of portomesenteric vein thrombosis after SG is 0.5-1% [1-3]. Although portal vein thrombosis after sleeve gastrectomy has been reported several times in the literature as a complication, splenic rupture due to portal and splenic venous thrombosis after SG is extremely rare and a life-threatening complication of SG [4, 5]. Here we present the first case, to our knowledge, of splenic rupture due to portal vein thrombosis after SG triggered by defecation.

## Case presentation

**Figure 1:**
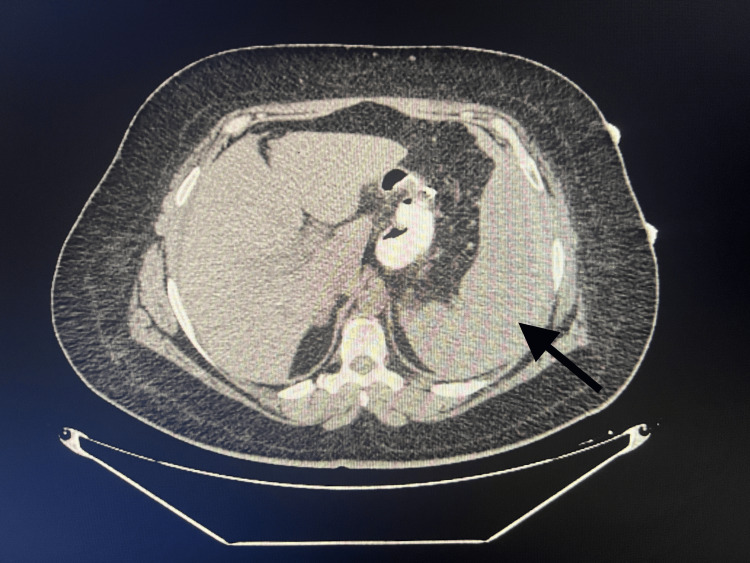
CT image of the patient on day of admission. Arrow indicates enlarged spleen.

A 38-year-old female patient presented to our emergency room complaining of acute abdominal pain, fatigue, and cold sweating for the past 36 hours. She had a history of a laparoscopic sleeve gastrectomy due to morbid obesity 13 days ago and she was discharged on a two-week course of a prophylactic dose of LMWH. The patient's pulse was 114 bpm and arterial blood pressure was 100/65 mmHg. Abdominal examination revealed diffuse tenderness but no rebound. She initially presented with a hemoglobin level of 10.8 g/dl. Portal system Doppler ultrasound showed increased periportal echogenicity, the portal vein was 10 mm in diameter and had an anechoic lumen with no flow, thus findings were consistent with portal vein thrombosis. Additionally, the CT scan revealed that the volume of the spleen was increased, its margins were regular and the density of the splenic parenchyma was normal (Fig. 1). The patient was monitored in the intensive care unit with LMWH treatment.

**Figure 2:**
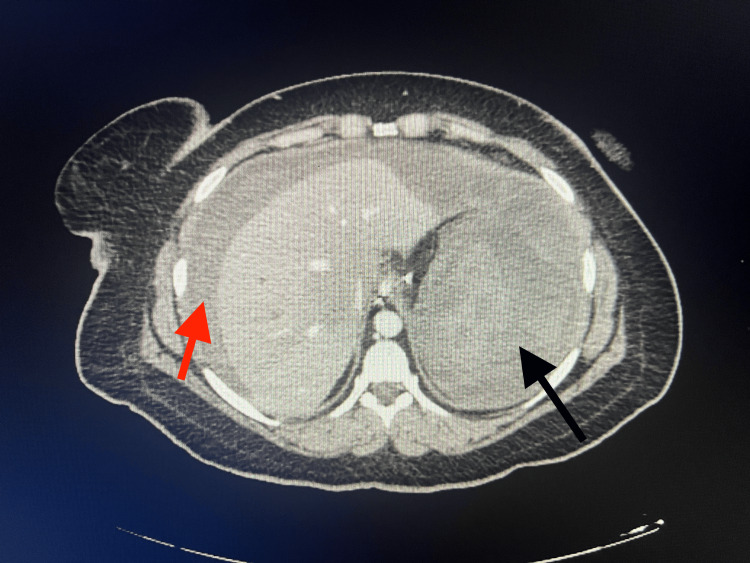
Splenic rupture with hemoperitoneum. The red arrow indicates intra-abdominal fluid due to bleeding, and the black arrow shows the ruptured spleen.

**Figure 3:**
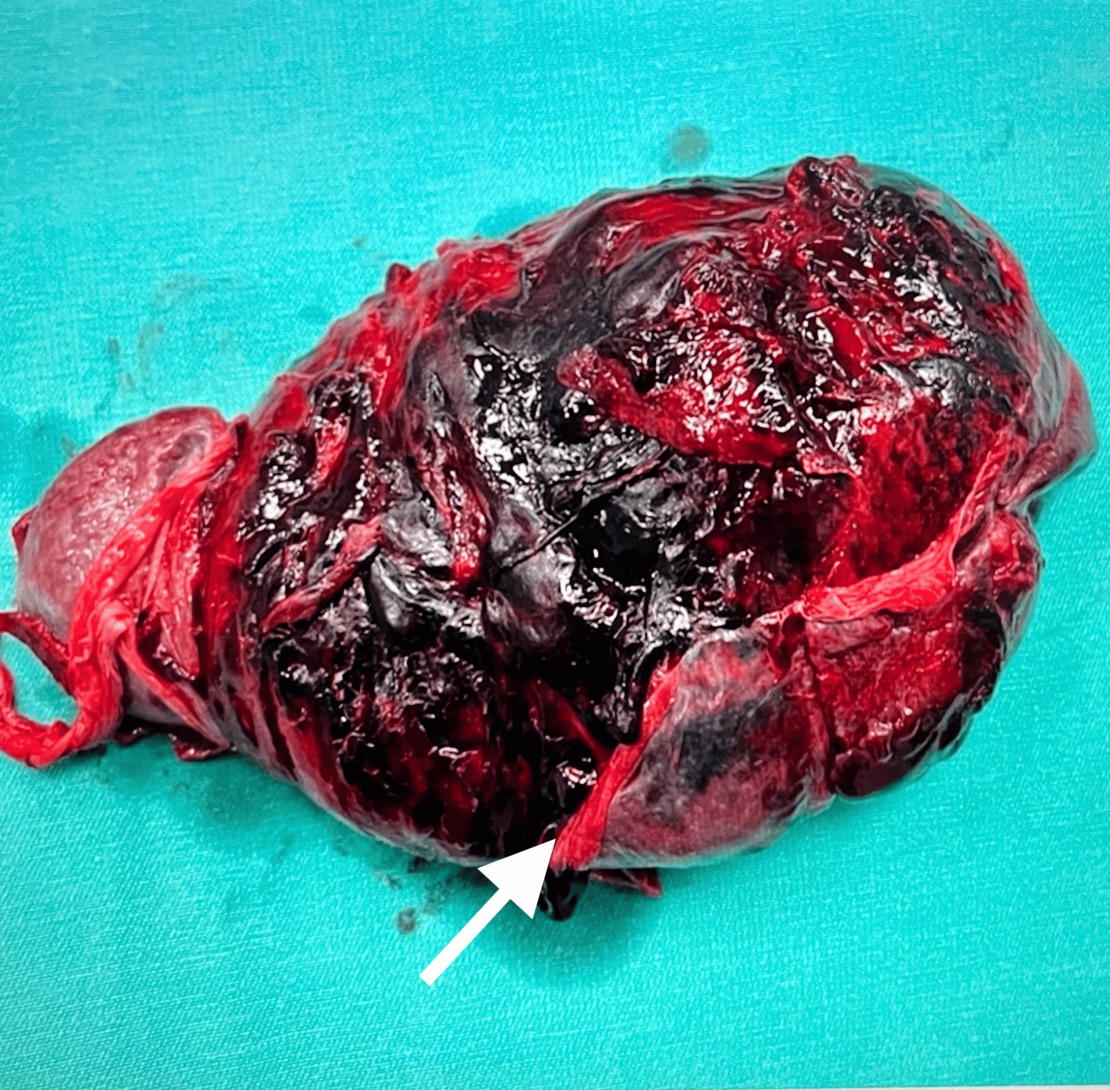
IImage of explanted spleen. The arrow indicates the margin of separation between the multiple splenic lacerations and the intact capsule.

While the patient's vital signs were stable on the second day of hospitalization, she was observed with complaints of severe abdominal pain and syncope immediately after defecation and her general condition deteriorated. Her heart rate was 160/min and her blood pressure was 80/55 mmHg. The Hb level decreased to 3.4 mg/dl (Table 1). The patient was transfused with six units of red blood cells as a bolus. IV contrast-enhanced abdominal CT showed hematomas undistinguishable from the parenchyma surrounding the splenic contour and diffuse hemorrhagic density values were observed in the abdomen, suggesting the presence of splenic rupture (Fig. 2). No contrast was seen in the portal and splenic veins. Emergency laparotomy was performed and 5 liters of hemoperitoneum, severe splenic hemorrhage, and splenic rupture were noted (Fig. 3). Splenectomy was performed and a drain was placed in the abdomen. A total of 11 units of red blood cells were transfused perioperatively. The patient was discharged on the 10th postoperative day uneventfully.

**Table 1:** Laboratory findings of the patient

Parameters	Admission	Day of splenic rupture	Reference values	Unit
White blood cell	12.25	18.42	4.5-11	x10^3^cells/mL
Hemoglobin	10.8	3.4	11.7-15.5	g/dl
Platelet	299	107	150-400	x10^3^/mL
Creatinine	0.91	1.07	0.5-0.9	mg/dl
C-reactive protein (CRP)	79.2	205.8	<5	mg/L

## Discussion

The development of portomesenteric vein thrombosis is an uncommon complication of SG [4]. The exact pathogenesis of PVT after sleeve gastrectomy remains unknown. Some of the pathogenic factors reported are prolonged retraction of the liver, thrombophilic state, inadequate antithrombotic prophylaxis, and delayed mobilization of the patient after surgery. Additionally, ligation of the gastroepiploic vessels during greater curvature dissection, high levels of pneumoperitoneum, and high intragastric pressure, are among the surgical factors [1, 6]. PVT has the potential to cause splenomegaly, portal hypertension, mesenteric ischemia, esophageal varices, and gastrointestinal bleeding. These complications can progress to life-threatening conditions. Therefore, it is very important for bariatric surgeons to inform the patient and closely monitor suspicious cases.

Case presentations of portomesenteric vein thrombosis may vary depending on the level of obstruction. The most common symptom that has been reported is abdominal pain which can be described as diffuse, vague, left or right, and can also radiate to the back. However, the presence of hematemesis, although uncommon, is one of the most urgent reasons to treat patients with portal vein thrombosis [7]. According to the literature, the first line of treatment for PVT should be anticoagulation therapy. Although it is possible and has been reported in some cases, spontaneous thrombus recanalization is extremely rare [8]. Recommendations to prevent this complication include limiting operative time, controlling values of the pneumoperitoneum, careful use of an energy device during dissection of the gastric greater curvature, and appropriate prophylaxis wıth low molecular weight heparin [7].

Splenic rupture after laparoscopic sleeve gastrectomy is extremely rare. To the best of our knowledge, there are seven cases of splenic rupture after sleeve gastrectomy in the literature, including our case. There were three cases of splenic rupture associated with venous thrombosis, including the cases presented by Salgado et al. [4], Gangemi et al. [5], and the present case. Spontaneous splenic rupture following sleeve gastrectomy has been documented in three case reports by Sandal et al [9], Manuela et al. [10], and Mertens et al. [11]. Humenansky et al. [12] explained splenic rupture as secondary to air autodissection caused by pneumoperitoneum. In six of these seven cases in the literature, treatment was achieved by splenectomy. Only Mertens et al. [11] successfully treated the patient with spleen-preserving surgery via a laparoscopic approach using hemostatic material (Table 2).

**Table 2:** Reported cases of splenic rupture following sleeve gastrectomy

Case reports	Age (year)	Gender	Cause of splenic rupture	Time after SG (day)	Management
Current case, 2024	38	Female	Portal vein thrombosis	15	Open splenectomy
Mertens et al., 2024 [11]	42	Male	Spontaneous	2	Laparoscopic spleen-preserving surgery
Sandal et al., 2020 [9]	32	Male	Spontaneous	14	Open splenectomy
Manuela et al., 2018 [10]	55	Female	Spontaneous	2	Open splenectomy
Humenansky et al., 2017 [12]	58	Female	Subcapsular gas collection	20	Open splenectomy
Gangemi et al., 2016 [5]	41	Male	Splenic vein thrombosis	7	Open splenectomy
Salgado et al., 2012 [4]	51	Female	Portal vein thrombosis	11	Open splenectomy

Interestingly, in our case, sudden clinical deterioration, severe tachycardia, and hypotension were noted immediately after defecation. Splenic rupture may have occurred due to stretching of the splenic ligaments by bowel movements during defecation. In addition, the increase in intra-abdominal pressure caused by defecation may have led to increased tension and rupture of the capsule surrounding the splenic hematoma. The case we present is the only case in the literature of splenic rupture triggered by defecation, with a history of PVT after SG. However, cases of splenic rupture caused by defecation have been reported in the literature in various situations [13, 14]. In a more unusual case, Kodikara and Sivasubramanium reported that the pressure exerted by a full stomach after a heavy and solid meal could rupture a perisplenic hematoma through a tear on the gastric surface of the spleen, causing delayed splenic rupture and sudden intraperitoneal hemorrhage [15].

## Conclusions

Splenic rupture is a life-threatening complication due to sudden, massive bleeding. In patients with portomesenteric vein thrombosis and/or splenic hematoma, conditions that cause tension in the abdominal ligaments and increase intra-abdominal pressure can lead to splenic rupture, as seen in the present case. A detailed evaluation of cases developing portomesenteric vein thrombosis after SG in the literature may provide remarkable and conclusive data to reduce morbidity and mortality rates after SG.
